# An mHealth Platform for People With HIV Receiving Care in Washington, District of Columbia: Qualitative Analysis of Stakeholder Feedback

**DOI:** 10.2196/48739

**Published:** 2023-09-19

**Authors:** Sylvia Caldwell, Tabor Flickinger, Jacqueline Hodges, Ava Lena D Waldman, Chloe Garofalini, Wendy Cohn, Rebecca Dillingham, Amanda Castel, Karen Ingersoll

**Affiliations:** 1 Center for Behavioral Health and Technology Department of Psychiatry and Neurobehavioral Science University of Virginia Charlottesville, VA United States; 2 Division of General Medicine, Geriatrics and Palliative Care Department of Medicine University of Virginia Charlottesville, VA United States; 3 Division of Infectious Disease and International Health University of Virginia Charlottesville, VA United States; 4 Milken Institute School of Public Health George Washington University Washingon, DC United States; 5 Public Health Sciences University of Virginia Charlottesville, VA United States

**Keywords:** HIV, mobile health, mHealth, cluster randomized controlled trial, formative, adaptation, qualitative methods, smartphone, mobile phone

## Abstract

**Background:**

HIV viral suppression and retention in care continue to be challenging goals for people with HIV in Washington, District of Columbia (DC). The *PositiveLinks* mobile app is associated with increased retention in care and viral load suppression in nonurban settings. The app includes features such as daily medication reminders, mood and stress check-ins, an anonymized community board for peer-to-peer social support, secure messaging to care teams, and resources for general and clinic-specific information, among other features. PositiveLinks has not been tailored or tested for this distinct urban population of people with HIV.

**Objective:**

This study aimed to inform the tailoring of a mobile health app to the needs of people with HIV and their providers in Washington, DC.

**Methods:**

We conducted a 3-part formative study to guide the tailoring of PositiveLinks for patients in the DC Cohort, a longitudinal cohort of >12,000 people with HIV receiving care in Washington, DC. The study included in-depth interviews with providers (n=28) at study clinics, focus groups with people with HIV enrolled in the DC Cohort (n=32), and a focus group with members of the DC Regional Planning Commission on Health and HIV (COHAH; n=35). Qualitative analysis used a constant comparison iterative approach; thematic saturation and intercoder agreement were achieved. Emerging themes were identified and grouped to inform an adaptation of PositiveLinks tailored for patients and providers.

**Results:**

Emerging themes for patients, clinic providers, and COHAH providers included population needs and concerns, facilitators and barriers to engagement in care and viral suppression, technology use, anticipated benefits, questions and concerns, and suggestions. DC Cohort clinic and COHAH provider interviews generated an additional theme: clinic processes. For patients, the most commonly discussed potential benefits included improved health knowledge and literacy (mentioned n=10 times), self-monitoring (n=7 times), and connection to peers (n=6 times). For providers, the most common anticipated benefits were improved communication with the clinic team (n=21), connection to peers (n=14), and facilitation of self-monitoring (n=11). Following data review, site principal investigators selected core PositiveLinks features, including daily medication adherence, mood and stress check-ins, resources, frequently asked questions, and the community board. Principal investigators wanted English and Spanish versions depending on the site. Two additional app features (messaging and documents) were selected as optional for each clinic site. Overall, 3 features were not deployed as not all participating clinics supported them.

**Conclusions:**

Patient and provider perspectives of PositiveLinks had some overlap, but some themes were unique to each group. Beta testing of the tailored app was conducted (August 2022). This formative work prepared the team for a cluster randomized controlled trial of PositiveLinks’ efficacy. Randomization of clinics to PositiveLinks or usual care occurred in August 2022, and the randomized controlled trial launched in November 2022.

**International Registered Report Identifier (IRRID):**

RR2-10.2196/37748

## Introduction

### Background

Given the high rates of new HIV diagnoses, Washington, District of Columbia (DC), is one of the priority jurisdictions for the US national Ending the HIV Epidemic initiative [1]. Among the various approaches to ending the HIV epidemic, the ability to engage people with HIV in care and help them achieve viral suppression may lead to a reduced rate of HIV transmission. Certain subpopulations are disproportionately affected by HIV within DC. These include people with HIV who identifies as men and Black, men who have sex with men, and those who are uninsured [2]. Various barriers to retention in HIV care have been identified specifically for people with HIV in DC, including transportation, a lack of comprehensive medical services (case management and adherence-related services), a lack of a patient-centered medical home, and gaps in health knowledge [3-7].

### The DC Cohort

The DC Cohort is the largest citywide prospective cohort of people with HIV in the United States [8]. Funded by the National Institutes of Health, the study is conducted in partnership with the DC Center for AIDS Research, the DC Department of Health, and the National Institutes of Health National Institute of Allergy and Infectious Diseases as part of the DC Partnership for AIDS Progress [6]. As of December 2021, there were 11,904 people with HIV living in DC, with an estimated 75% of DC Cohort participants being DC residents [9]. The DC Cohort is representative of the broader HIV population in the DC area, with 82% (n=4258) of participants identifying as Black, 68% (n=3533) identifying as man, and 38% (n=1977) being men who have sex with men in 2017 [10].

### Mobile Health Apps for HIV

As of 2017, approximately 325,000 mobile health (mHealth) apps are available [11]. A growing evidence base demonstrates that mHealth apps can have a positive impact on health [12,13]. Several mHealth interventions have been developed to support self-management and provide social and mental health support for people with HIV, and some of these interventions have been associated with improved antiretroviral therapy adherence [14-21]. *PositiveLinks* is a clinic-deployed mHealth app designed for people with HIV with the goal of improving connection to HIV care and peers with HIV [22]. PositiveLinks was developed based on psychological theories of behavior change [23,24] and user-based design principles and practices [25-28]. PositiveLinks features include self-monitoring of daily medication adherence, mood and stress “check-ins” with a calendar display for retrospective review, an anonymized community board for peer-to-peer social support and connections, Health Insurance Portability and Accountability Act–compliant private messaging between provider and patient, a document upload feature to share Ryan White eligibility and care-related documents between clinic staff and the patient, appointment reminders, weekly quizzes, a clinic-specific contact directory, and features with general HIV education and local resources [28,29]. PositiveLinks use has been associated with improved engagement in care and HIV viral suppression and increased cluster of differentiation 4 counts across several studies [22,28,29]. In 2022, PositiveLinks was named an evidence-based program or practice to improve engagement in care for people with HIV by the Health Resources and Services Administration [30].

### Purpose of Formative Research

Formative studies and user-centered design processes with end users can identify features, inform the look and feel, and improve the usability of a mobile app for the population [31-33]. Previous research on clinic deployment of PositiveLinks has been conducted in nonurban clinics [24,34]. We anticipated different demographics and unique challenges of people with HIV living within the DC area [5,6], where PositiveLinks will be implemented among DC Cohort clinics in the first large cluster randomized controlled trial, testing its efficacy against usual care and studying the implementation of the program. A specific aim of this study was to identify app adaptations needed to tailor PositiveLinks to be feasible, acceptable, and usable for people with HIV in the DC area. Furthermore, we aimed to identify logistical considerations related to implementing PositiveLinks as part of clinical care at DC Cohort sites participating in the trial [7]. Finally, we anticipated that providers and patients using the app would have differing needs that might affect app tailoring and that tailoring of program training would need to maximize usability and acceptability for both groups of end users. Therefore, we included patient and provider perspectives in formative studies.

## Methods

### Overview

We conducted focus groups (FGs) with a sample of 32 people with HIV enrolled in the DC Cohort. We conducted in-depth interviews with 32 providers from 16 DC Cohort clinics. Owing to the size of the DC Regional Planning Commission on Health and HIV (COHAH), this group (n=35) was divided into 2 to allow for better data capture. In total, 2 COHAH FGs took place on the same night via Zoom (Zoom Video Communications). Providers included any DC Cohort clinic staff providing services to patients, including physicians, nurse practitioners, physician assistants, clinical nursing staff, social workers, case managers, and administrative staff. Informed consent was obtained from all participants per the institutional review board–approved protocol.

### People With HIV Enrolled in the DC Cohort

#### Recruitment

Clinic site research assistants recruited patient participants from each of the 14 DC Cohort clinics. Research assistants conducted both in-person and telephone recruitment of potential patient participants. Patients were approached in person when attending medical visits at the participating clinics. Patients who expressed interest were given the contact information for a study team member. The study team member screened interested patients for eligibility. Patient eligibility criteria included being aged ≥18 years; being English speaking; receiving care at 1 of the 14 DC Cohort clinics; and being able to participate on a web-based platform, such as Zoom, because of COVID-19 restrictions. Participants were remunerated for their time with a US $25 gift card.

#### Patient FGs

Patient FGs took place from June 2021 to August 2021. In total, 32 patients participated in the FGs. A total of 5 FGs were held, with each group having 5 to 8 participants for a total of 32. All patients who were referred and met the eligibility criteria provided informed consent. Patients were asked to complete a REDCap (Research Electronic Data Capture; Vanderbilt University) survey providing demographic information before attending the FG. If a patient had difficulties completing the survey before the scheduled FG, a study team member worked with them after the FG to ensure completion of the survey. All patient FGs were conducted on Zoom and recorded. To protect privacy, each patient was given an unrelated nickname to use during the FG. Patients were not required to use their cameras during the FG but could if they chose. The FGs were semistructured and followed interview guides with sections on patient knowledge of concepts, including retention in care, viral suppression, and barriers to and facilitators of retention in care and viral suppression. Patients were asked about their use of technology and mHealth interventions. FG participants then watched a short video demonstration of the PositiveLinks app. Following the demonstration, patients provided feedback about the app interface, any potential concerns related to future implementation at the site where they received care, and usability of the app from the perspective of a person with HIV in the DC-Maryland-Virginia area. They commented on the app’s utility to facilitate retention in care and viral suppression and shared general thoughts and suggestions for improvement.

### DC Cohort Site Providers and the COHAH

#### Recruitment

Providers were recruited for the in-depth interviews via an email from the study team staff in collaboration with site principal investigators (PIs). To be eligible to participate, the provider had to speak English and work in a patient-facing position at a DC Cohort clinic.

#### Provider In-Depth Interviews

Provider in-depth interviews took place from January 2021 to August 2021. In total, 2 providers from each of the 14 DC Cohort clinics participated in in-depth interviews (n=28). Informed consent was obtained verbally from all participating providers. All providers were sent the informed consent form before the interview session. During the interviews, a demonstration of the PositiveLinks app was given to provide a baseline understanding of the app and prompt provider discussion about their perceptions of it and its potential use in their clinic. All interviews were conducted over Zoom and recorded. The REDCap sociodemographic survey was emailed to providers and completed before the interview sessions.

Semistructured interview guides included questions about the characteristics of the population served, existing clinic processes, workflow, barriers and facilitators related to patient retention in care and viral suppression, and provider knowledge or experience with mHealth interventions or digital tools used in their clinical work. Participants were provided with a demonstration of the PositiveLinks app. Following this, the providers shared their thoughts about the app interface, potential usability of the app in their patient interactions, and concerns related to future implementation at their site. Providers were also asked for open-ended feedback and opinions about the app with respect to patient care.

### FGs With the COHAH

A total of 2 FGs were held with the COHAH. All members of this commission provide direct care through various roles to people with HIV in the DC-Maryland-Virginia area. Participation in the FG was offered to all current commission members. These FGs took place in April 2021 during a regularly scheduled COHAH meeting and included 35 members. The eligibility criterion for these participants was being English speaking. The study team provided an app demonstration to participating COHAH members. After the demonstration, participants joined 1 of 2 FGs meeting concurrently over Zoom. The session moderators followed the same semistructured guide and format as had been used for the provider interviews. All members were sent an informed consent form and the REDCap sociodemographic survey via email before the meeting. Informed consent was provided before the FGs. REDCap surveys that were not completed before the FGs were completed afterward.

### Data Analysis

Individual interviews and FGs were audio recorded and professionally transcribed verbatim. To ensure privacy and confidentiality, participants’ names were not used during the interviews. If a name was mentioned, it was removed during transcription. Deidentified transcripts were uploaded to Dedoose for qualitative analysis (version 8.0.35; SocioCultural Research Consultants) [35]. Coders included 5 members of the research team. Coders had varied educational backgrounds, including Medical Doctorate, Doctorate of Health Science, Masters of Public Health, and Masters of Science. All coders had previous experience with qualitative coding. None of the coders had relationships with the participants before the FGs. A total of 4 different interviewers facilitated the FGs depending on their availability. One coder also facilitated FG interviews. Coders jointly developed a codebook for the provider interviews using an open coding approach and constant comparison methodology.

The initial codebook included deductive codes informed by the interview guide to capture anticipated codes for PositiveLinks features and clinic characteristics and processes. It also included inductive codes derived from FG participants’ perspectives to generate emerging themes. The codebook was refined through an iterative process with at least 2 members of the study team independently coding each interview and resolving any discrepancies through consensus until thematic saturation and acceptable intercoder agreement were achieved. The COHAH FG transcripts were included with the provider interviews in 1 data set and coded using the same codebook. After provider data coding was completed, the codebook was adapted for use with the patient FGs. Additional emerging themes were included to capture the patient perspective for thematic saturation from the patient FGs. Each patient FG was coded by 2 members of the study team, and a consensus was reached. We achieved 70% intercoder agreement on provider interviews and 72% agreement on patient FGs.

For provider and patient data, the codebooks were applied to the entire data set so that code frequencies could be determined. For FG transcripts, it was not always possible to distinguish which individual participant was speaking. Therefore, code frequencies are reported as the number of instances in which a code was applied, not the number of participants who mentioned each code. Within each category of codes (eg, barriers to engagement in care), we assessed the most commonly occurring themes and representative examples. Coders discussed the potential impact of themes on PositiveLinks implementation in the DC Cohort trial and possible action steps for research staff to address them. We compared themes from the provider and patient perspectives and implications for the optimization of the PositiveLinks app for both stakeholder groups. A summary of the findings was provided for the trial study team to inform their selection of specific app functionalities, additional functionalities for development, and considerations for the implementation of PositiveLinks and associated program activities at participating sites.

### Ethics Approval

All study activities were reviewed and approved by the George Washington University Institutional Review Board and external institutional review boards (protocol NCR202829; ClinicalTrials.gov NTC04998019).

## Results

### Patient FGs

#### Overview

Baseline demographic information for participating people with HIV is summarized in Table 1. On average, participants were aged 53 (SD 11.26) years, with ages ranging from 27 to 73 years.

**Table 1:** Baseline demographic data of patients participating in focus group sessions (n=32).

Characteristic			Values
Age (years), mean (SD)			53 (11.26)
Gender identity (women), n (%)			18 (56)
**Race^a^, n (%)**			
	Asian	1 (3)	
	Black or African American	29 (91)	
	White	4 (12)	
**Current residence, n (%)**			
	Washington, DC^b^	28 (88)	
	Maryland	4 (12)	
	Virginia	0 (0)	
**Highest education level completed, n (%)**			
	<Grade 8	0 (0)	
	Grades 9-11	4 (12)	
	Grade 12 or GED^c^	8 (25)	
	Some college, associate degree, or technical degree	12 (38)	
	Bachelor’s degree	5 (16)	
	Any postgraduate studies	3 (9)	
**DC Cohort enrollment clinic, n (%)**			
	Whitman-Walker Institute	9 (28)	
	Unity Health Center	5 (16)	
	MetroHealth	4 (12)	
	Georgetown University Medical Center	4 (12)	
	Family and Medical Counseling Service	3 (9)	
	Howard University—adult	3 (9)	
	Howard University—pediatrics	2 (6)	
	Washington Health Institute	2 (6)	
**Types of mobile apps used, n (%)**			
	Games and entertainment	30 (94)	
	Social media	29 (91)	
	Lifestyle	27 (84)	
	News and information	24 (75)	
	Utility	24 (75)	
	Productivity	11 (34)	
**Provider engagement^d^, n (%)**			
	In person	22 (69)	
	Telephone call	13 (41)	
	Video call—phone	15 (47)	
	Phone app	5 (16)	
	Video call—computer	2 (6)	
	Computer application	0 (0)	

^a^Participants could select more than 1 race.

^b^DC: District of Columbia.

^c^GED: general educational development.

^d^Participants were asked how they engaged with their provider in the past year (during the COVID-19 pandemic).

#### Themes

##### Overview

Several major themes emerged from the qualitative analysis of patient FGs. Themes were grouped into population needs and concerns, barriers to and facilitators of engagement and viral suppression, technology use, anticipated benefits associated with PositiveLinks, and patient concerns. All textboxes display the top 3 themes as ranked by their frequency of mention by participants during FG discussions.

##### Population Needs and Concerns

The most common patient needs and concerns stated by participants were low health literacy, mental health needs, and medical comorbidities. Several participants (6/32, 19%) mentioned having difficulty understanding aspects of their health because of low health literacy. One participant stated the following:

I’m still trying to learn how to actually read them...I’ve always had an issue reading my viral load.

Mental health needs were expressed 5 times by participants, specifically the concern of not seeking help for mental health–related problems. As one participant stated, “Some people don’t want to acknowledge they have mental health issues. I’ve been in that place before as well so it’s like they’re just trying to manage the best they can without help.” In total, 9% (3/32) of the participants also expressed concerns about managing medical comorbidities along with their HIV care.

##### Facilitators of and Barriers to Engagement and Viral Suppression

A variety of facilitators of and barriers to engagement and viral suppression were described by patient participants (Textbox 1). The most common facilitators of engagement and viral suppression were consistency with care (n=35), having a positive relationship with the clinic team (n=23), and patient empowerment (n=18). Other facilitators identified included social support (n=9), care coordination (n=6), and having a reminder or alarm for medications and health care appointments (n=3). The most common barriers to engagement and viral suppression were mental health (n=7), substance use (n=5), and stigma (n=4). Twice participants mentioned competing priorities as a barrier to engagement in care and viral suppression. For example, a participant mentioned having to prioritize housing and food security over taking their medications regularly and attending clinic appointments.

**Facilitators of and barriers to patient engagement in care and viral suppression that emerged during patient focus groups.**

**Facilitators**

- Consistency in care (n=35): “And it’s like getting the reward that you’re seeking and you want to just keep on going but you know it’s a challenge to just go day after day after day, do what you’re supposed to do, do what you should do, just stay on track.”
- Positive relationship with clinic team (n=23): “I think for me the most important thing that I look for in any physician that I deal with is their ability to connect with me but also have a sense of compassion. I love being with physicians who are not there just to get a check and to count numbers...but a person who sincerely looks out for me as an individual not just as a patient, a number, a status or whatever but as an individual who wants to see me thrive and do better for myself...”
- Empowerment (n=18): “Like I told my husband I’ve got to be my own facilitator, you know spokesperson, so I got on the phone and I got to ordering me this, I got to ordering me that. I had to take care of myself because wasn’t nobody going to step in and come and do it for me.”

**Barriers**

- Mental health (n=7): “Yes sometimes you have to educate people because there is still a lot of stigma it’s just like mental health. There’s a lot of stigma about mental health and it doesn’t need to be but there is so the same thing for HIV. I put that in almost the same kind of category really.”
- Substance use (n=5): “...when I was first diagnosed positive I was on drugs and my viral load was high. And I never knew why it was high but I just know I kept being sick, I was losing weight and I knew that me drugging, not getting the proper rest, running the street, that played a lot of me not taking my medication like I was supposed to.”
- Stigma (n=4): “You know what? I am my own barrier because I always thought that I would never find somebody who would love me for me. I used to have problems with that.”

##### Technology Use

A variety of technological apps were mentioned by participants. The most frequently mentioned technologies related to the participants’ health were health apps not associated with their clinic’s electronic medical record (EMR; n=25), for example, apps that track members’ weight, food or calories, and physical activities. Some participants stated that they used more than one health app. EMR-associated patient portals were the second most mentioned technology used by participants (n=7). Various patient portals were mentioned as the different clinics use different EMRs. Several patient participants expressed disinterest in using available patient portals (n=3):

...and the patient portal I don’t use very much, I’m a phone person.

Participants also discussed the advantages (n=13) and disadvantages (n=11) of the current technology they used for their HIV care. Advantages included the accessibility of information, the ability to correct information, the ability to access many features in one place, the ability to connect to the care team, reminders, and overall convenience. For example, a participant said the following:

...you can do just about all sorts of things, even request special appointments from doctor visits to dental to vision to even getting referrals. If you lost your referral, you can go back on there. You can [re-pull] documents that they’ve done sent you. You can see not only your lab results, you can see medical records.

Although some participants saw the accessibility and completeness of information in current technology as an advantage, others had a less positive experience. Some had difficulty understanding information as it was presented in the portal they used and perceived that most apps and portals were not comprehensive, which were cited as disadvantages. For example, a participant expressed issues with attempting to use their current technology, stating that “I try to use it, I can’t get to it.” Participants also expressed a desire for better and broader communication with the clinic.

##### Anticipated Benefits and Questions or Concerns

Participants discussed the anticipated benefits and concerns associated with using PositiveLinks (Textbox 2). The most common feedback on PositiveLinks was a general positive impression (n=30). For example, a participant stated that “I love the whole package.” Of the specific anticipated benefits discussed, the most common was improved health knowledge and literacy (n=10). Other most mentioned anticipated benefits were self-monitoring (n=7), connection to peers (n=6), and ease of use (n=6). Additional benefits mentioned by participants included connection to care. A participant stated that “I think you can really use this app to better connect yourself with your doctors and your case managers.” In addition, participants cited the reminder function as providing an anticipated benefit. Improved communication with the clinic team, empowerment or self-efficacy, and a reduction in stigma were also stated as anticipated benefits.

**Anticipated benefits and additional questions or concerns brought up during patient focus groups.**

**Anticipated benefits**

- Improved health knowledge or literacy (n=10): “So if they get the apps in these clinics and then people can learn stuff and don’t be ashamed then they can come in and start taking their medication and everything then they’ll take the information to other people.”
- Self-monitoring (n=7): “I like *How Am I?*, that’s the one where you log in where you take your medicine ‘cause sometimes I may take my medicine and I may forget it and I’m like ‘did I take my medicine?’ I can’t remember. And I’m like I should write it down but I keep forgetting to write it down. And sometimes I do miss my medications, I can’t remember if I took it or not and I don’t want to do too much.”
- Connection to peers (n=6): “I think it would help them especially in the Community Board group because you can put anything you really want to ask somebody out there and not from a doctor’s perspective but from people that are actually living it.”
- Ease of use (n=6): “I will say it seems really easy to navigate...It didn’t seem like it’s going to be a hard app.”

**Questions or concerns**

- Possible redundancy (n=5): “I said I think so because it’s very similar to (other app). And what I have read on there its helpful and y’all might have a little bit more information to add to it but it’s similar.”
- Lack of coordination with non-HIV care (n=5): “It would be useful to have a way for the people who have a medical home to go to other hub...to support services. They’re linked but it would be important to link them through this system if at all possible.”
- Privacy or security (n=5): “...speaking about the app that’s kind of touch and go because that sensitive information you’re speaking about trusting it on an app...”

The issue of privacy was both an anticipated benefit and a concern. A participant who viewed the privacy of the app as a benefit stated the following:

That’s wonderful, yeah that’s perfect. Because now-a-days when you save a picture it’s not only on your phone it’s in your Google photos and it’s all over the world.

This participant was referring to the document function, which allows a user to upload a document to their provider using their phone’s camera but does not store the picture on the phone itself, only in the app behind log-in protections. Others viewed privacy and security as a potential concern, primarily related to accidental disclosure of diagnoses or related personal health information.

In addition to privacy and security, the 2 most mentioned questions and concerns about PositiveLinks were possible redundancy with other apps (n=5) and potentially a lack of coordination with non-HIV medical care (n=5). Low technological literacy was also mentioned as a concern (n=4). A participant noted the following:

...some people don’t know how to use the phone or any technology to get on any kind of app to learn anything. Some people just use the phone to talk with.

Similarly, it was twice mentioned that participants thought the phone app was difficult to use, specifically stating that they preferred to use a computer as the phone app font was too small and that they had a hard time pushing buttons on their phone.

##### Suggestions

During the FGs, participants made suggestions for improving PositiveLinks. Some of the suggestions included adding a virtual video support group feature (n=6) and more resources (n=5), such as an in-app DC Metro schedule. Patients suggested additional reminders (n=3), such as reminders for all their medicines and appointments, not just HIV-specific reminders, as well as including additional tracking functions (n=3), allowing for more comprehensive self-management of overall health and well-being. The importance of patient training to use the app was also mentioned by 9% (3/32) of the participants, with one suggesting that hour-long classes might be needed to review the app step by step with newly enrolled members.

### Provider In-Depth Interviews and FGs

#### Overview

Baseline demographic information for participating providers is summarized in Table 2. Most participating providers were aged ≥55 years (10/29, 34%); did not identify as Hispanic, Latino, or Latina (24/29, 83%); and identified as White (15/29, 52%). More than half (15/29, 52%) of the participating providers were medical doctors, whereas the remainder included social workers, nurses, and other roles. The number of years caring for people with HIV ranged from <5 (5/29, 17%) to ≥20 (10/29, 34%).

**Table 2:** Baseline demographic data for providers and District of Columbia Regional Planning Commission on Health and HIV (COHAH) members participating in in-depth interviews and focus groups.

Characteristic			Providers (n=29)		COHAH members (n=35)
**Age group (years), n (%)**					
	25-34	4 (14)		8 (23)	
	35-44	6 (21)		7 (20)	
	45-54	9 (31)		3 (9)	
	≥55	10 (34)		16 (46)	
	Declined to answer or blank	0 (0)		1 (3)	
**Gender identity, n (%)**					
	Woman	19 (66)		13 (37)	
	Man	10 (34)		20 (57)	
	Declined to answer or blank	0 (0)		2 (6)	
**Ethnicity, n (%)**					
	Non-Hispanic	24 (83)		32 (91)	
	Hispanic	5 (17)		3 (9)	
**Race (multiple answers allowed), n (%)**					
	Asian	3 (10)		0 (0)	
	Black	9 (31)		22 (63)	
	Native American	0 (0)		2 (6)	
	White	15 (52)		13 (37)	
	Do not know	0 (0)		1 (3)	
	Declined to answer or blank	2 (7)		0 (0)	
**Degree, n (%)**					
	MD^a^	15 (52)		2 (6)	
	MSW^b^	5 (17)		3 (9)	
	RN^c^	1 (3)		1 (3)	
	PhD^d^	0 (0)		4 (11)	
	Other	8 (28)		23 (66)	
	Declined to answer or blank	0 (0)		2 (6)	
**Number of years caring for people with HIV,** **n (%)**					
	<5	5 (17)		5 (14)	
	5-9	5 (17)		11 (31)	
	10-14	6 (21)		8 (23)	
	15-19	3 (10)		4 (11)	
	≥20	10 (34)		5 (14)	
	Declined to answer or blank	0 (0)		2 (6)	
**Clinic services (multiple answers allowed), n (%)**					
	Linkage to care services	26 (90)		24 (69)	
	Primary medical care	25 (86)		14 (40)	
	Treatment and medication adherence services	25 (86)		20 (57)	
	HIV counseling and testing	25 (86)		22 (63)	
	HIV prevention services	24 (83)		23 (66)	
	Case management	23 (79)		17 (49)	
	Retention in care or navigation services	23 (79)		18 (51)	
	Support groups	15 (52)		17 (49)	
	Other	5 (17)		13 (37)	
	None	1 (3)		2 (6)	

^a^MD: Doctor of Medicine.

^b^MSW: Master of Social Work.

^c^RN: registered nurse.

^d^PhD: Doctor of Philosophy.

#### Themes

##### Population Needs and Concerns

The most frequently mentioned needs and concerns were mental health care (n=20), transportation (n=18), and housing (n=18). Additional concerns included limited access to phones (n=15) and Wi-Fi or data plans (n=10). Providers also expressed concerns about insurance eligibility (n=14), citing that “...we often see a lot of people who don’t have insurance who have lower incomes and or inconsistent or unstable employment and that represents a significant portion.” Providers also expressed concerns regarding food access and security (n=10).

##### Clinic Processes

Providers described their current processes for promoting client engagement and viral suppression. These included outreach to clients (n=24):

We e-mail patients and then after three attempts we send a letter to the address on file, just very discreet like hello...I just wanted to contact you to get you an appointment with us. Call me back at this number. It’s very HIPAA compliant and very simple. Yeah that’s our last step usually is sending a letter in the mail.

Tracking and surveillance strategies (n=24) included the following:

...in addition to the monthly QI meeting we also have a monthly HIV QI subcommittee that meets every month and so we go over that as well. And we do go over viral loads during those meetings.

Providers also discussed retention teams who performed outreach (n=20).

##### Facilitators of and Barriers to Engagement and Viral Suppression

Providers discussed what they perceived as facilitators of and barriers to engagement and viral suppression among their patient populations (Textbox 3). The most mentioned facilitators were patients’ positive relationships with the clinic team (n=15), transportation assistance (n=15), and other facilitators that helped with clinic access. Social support was mentioned 6 times as a facilitator, with a participant stating that “...they started hearing that other kids were having similar issues, they didn’t feel alone.”

**Facilitators of and barriers to engagement and viral suppression that emerged during provider interviews and District of Columbia Regional Planning Commission on Health and HIV focus groups.**

**Facilitators**

- Positive relationship with clinic team (n=15): “It’s when the staff shows that they do care about that person...I think that keeps them coming back.”
- Transportation assistance or food access (n=15): “We offer tokens for patients for transportation and vouchers for meals.”
- Integration or coordination of services (n=13): “...they address the housing, transportation, food...drug programs and also the psychiatric issues.”

**Barriers**

- Stigma or privacy (n=21): “She told us it was about the stigma. And a good amount of the stigma is internalized.”
- Low health literacy (n=19): “...health literacy is very low in general populations particularly populations we serve, just overall health literacy, forget HIV.”
- Mental health barriers to care (n=15): “...needing to navigate a system they’re not familiar with while depressed becomes a reason people might drop out of care.”

The most frequently mentioned barriers were stigma or privacy-related issues (n=21), low health literacy (n=19), and mental health issues (n=15). Unstable housing (n=12) was also listed as a barrier to engagement and viral suppression as “...housing is so incredibly difficult in this area.” Other providers cited patient insurance coverage (n=11), competing priorities (n=10), and financial challenges (n=8) as barriers. Additional barriers mentioned included substance use (n=12), transportation (n=7), transitions in care (n=7), distrust in providers (n=6), and lack of social support (n=4).

##### Technology Use

Telemedicine (n=22) was the most frequently mentioned form of technology use, followed by the use of non-EMR apps (n=17). These apps were primarily used for provider-patient communication and appointment and medication reminders. EMR patient portals (n=15) were also mentioned by providers.

Providers were also asked about the advantages and disadvantages of the technology currently used in their clinics. Advantages (n=11) included enhanced convenience in supporting tasks related to patient care, the care team’s connection to patients, perceptions of improved patient adherence to medications, EMR integration, and access to personal medical information. Disadvantages (n=12) included slow response time to messages, provider time commitment, and annoyance with the number of reminders received. Providers characterized ease of use for both patients and providers as well as the current level of security as both advantages and disadvantages.

##### Anticipated Benefits and Questions or Concerns

When considering PositiveLinks at their clinics, providers most frequently identified anticipated patient benefits, including improved communication with the clinic team (n=21), enhanced connection to peers (n=14), and facilitation of self-monitoring (n=11; Textbox 4). Patient empowerment and self-efficacy (n=7) were also mentioned as potential benefits, with a provider stating that “I think...especially the people who love their phones and their apps I think it would be great for them and lead toward their independence.”

The top 3 concerns cited by providers were related to patients’ potential technological difficulty or technological literacy (n=13), provider and administrator time commitment, and possible redundancy (n=11) with other technology use or clinic processes. Providers also expressed concerns about the liability associated with a platform such as PositiveLinks, with a provider asking, “...if something urgent were to come across this app and we didn’t see it who’s then going to be responsible or accountable?” Privacy and security were mentioned as both an anticipated benefit (n=10) and a concern (n=9). Providers specifically expressed concerns with communications related to patient care being housed outside the clinic’s EMR (n=5). Although not mentioned by most providers, those who expressed this concern had strong feelings about this topic.

Concerns related to the implementation of PositiveLinks were also discussed with providers. The top 3 concerns related to implementation were general concerns (n=12), alignment with current clinic workflow and existing processes or systems (n=4), and institutional approvals and permissions (n=4). Issues related to patient access to smartphones and data plans (n=4) that could support PositiveLinks were also mentioned, as stated by one provider:

Obviously a lot of times with our patients who are having difficulty with compliance, a lot of times they may not have phones or they may only have [basic] phones or they might not have data. And so sometimes that presents an issue.

**The *anticipated benefits* and *questions and concerns* themes that emerged from the provider in-depth interviews and District of Columbia Regional Planning Commission on Health and HIV focus groups.**

**Anticipated benefits**

- Improved communication with clinic team (n=21): “...it would just create a different communication realm than what’s available right now and maybe less burdensome for some people and I think that would be advantageous for them.”
- Connection to peers (n=14): “I think having an app that offers them the possibility to talk to other people who are in the same situations or share the same issues is going to help them, that would be great for them.”
- Self-monitoring (n=11): “...if I’m visually seeing if my viral load is going up and down then I’m going to be more involved in my care like oh my gosh actually I saw that last month I was low and now I’m high, now I’m going to do my best to get low again.”

**Questions and concerns**

- Technological difficulty or literacy (n=13): “It’s just not having the phone it’s literally technology and living in a world where technologically you are way behind and there’s a level of intimidation that is present as well as fear.”
- Provider or administrator time commitment (n=13): “...a lot of times when we’re adding new things it’s a question of the burden upon that staff member whether it’s a provider or case manager or whoever.”
- Possible redundancy (n=12): “...right now we’re getting messages in e-mails, we’re getting messages in the portal, we have voice mail, it’s just another platform that we have to be tuned into.”

**Implementation-related thoughts or concerns**

- General concerns (n=12): “I think I have positive things but implementing it and then how many of our patients would really be using it is a big big question.”
- Alignment with clinic flow (n=4): “If I already have a well-functioning system in the clinic how is it going to overlap or parallel that? For example we call each patient the day before an appointment in person or text them, individual case managers do reminders. And how will you account for the study the ongoing level of interaction and parallel to your app interaction? So that is to me an issue...And if it is a voluntary addition that’s fine but if it again a double system for example the notification thing will the patients get frustrated with us coming to them from every possible angle.”
- Institutional approvals or permissions (n=4): “Like really the only issue I foresee is [site] is extremely restrictive in terms of sharing patients’ information or communicating with patients outside of their channels.”

##### Suggestions

Providers were also asked for any suggestions they might have regarding PositiveLinks. The top 3 responses were including additional educational resources (n=7), ensuring that the app was very user-friendly (n=5), and building additional reminders into the app (n=4). Several of the provider participants suggested adding resources specific to each clinic and location on the resource tab in addition to larger regional or national resources. For example, it was suggested that each clinic site that uses PositiveLinks could have their clinic-specific forms available via the app for easy access.

**Figure 1:**
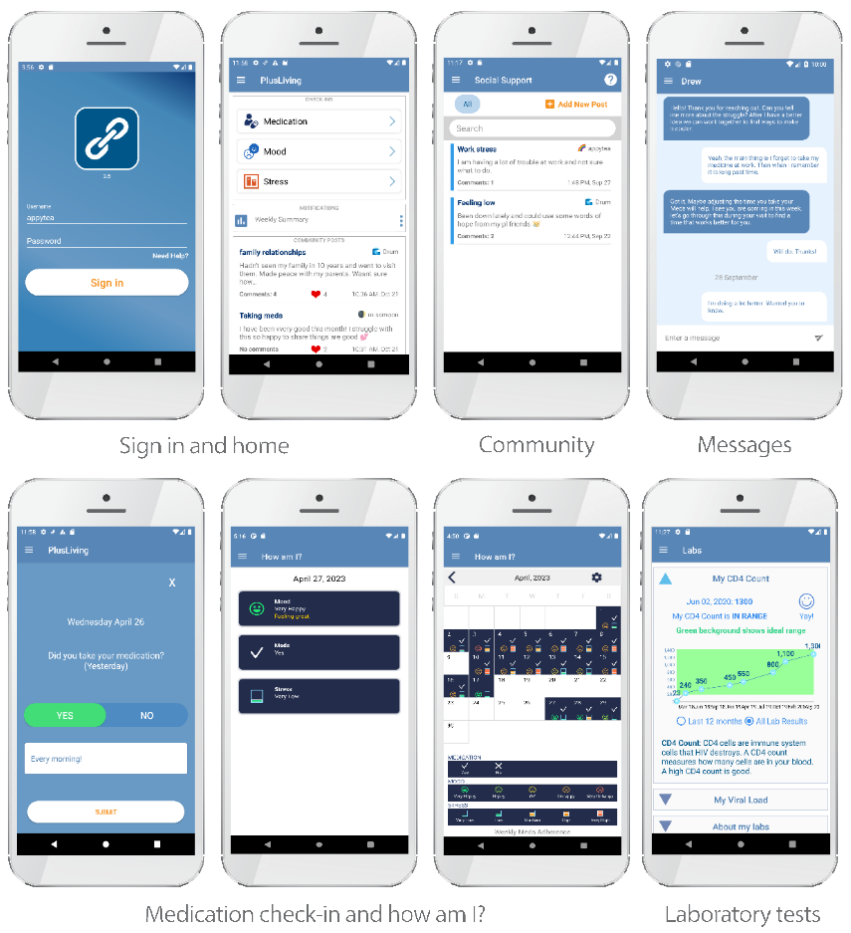
Screenshots of selected PositiveLinks features. Features shown include sign-in and home screens, medication check-in and How am I? pages, laboratory results page, community board main screen and conversation screen, and private messaging.

After completion of the patient FGs and provider interviews, a list of potential features to include in the rebuilding of the app for implementation within the randomized controlled trial was generated following review by the study team and then compiled and reviewed with the DC Cohort site PIs. The site PIs, along with the study team, chose to include the following features (Figure 1): mood, stress, and medication adherence check-ins; the *How am I?* check-in review calendar; laboratory tests (HIV viral load and cluster of differentiation laboratory values); community; resources; and frequently asked questions. The document and private messaging features were also selected to be part of the build but with the individual sites deciding whether they would use either of these functions. Appointment reminders and in-app telemedicine features were excluded from the build as formative work revealed redundancy with systems currently in use at several clinics.

## Discussion

### Principal Findings

We sought to elicit multistakeholder perspectives on how to adapt the PositiveLinks app to the urban people with HIV context. We found that stakeholder interviews, FGs, and stakeholder perceptions of the proposed PositiveLinks app generated action items to help tailor the platform to meet patient needs and identify logistical considerations related to the implementation of the PositiveLinks program. These data helped us optimize app usability for providers and inform program rollout and provider training at each participating Cohort site.

### Stakeholder Perspectives

Previous use of PositiveLinks has been associated with positive health outcomes such as viral suppression, a decrease in perceived stigma, and an increase in the feeling of social support [22,36]. This formative work demonstrated that PositiveLinks could promote existing facilitators for patient engagement identified by stakeholder groups by assisting with connection to care, building positive relationships with the clinic teams, increasing social support with peers, and improving health literacy and self-management. Patients and providers thought that PositiveLinks could be used as a means to address and potentially mitigate identified barriers, for example, (1) by confidentially connecting patients to members of the care team, such as case managers and social workers; (2) disseminating site-specific, regional, and national resources, such as services to assist with mental health challenges and substance use; and (3) reducing stigma through sharing accurate and up-to-date information, real-time encouragement, and facilitation of supportive peer interactions. These findings in an urban population in the United States align with previous PositiveLinks research in rural and suburban populations, suggesting that these features may be desirable to end user groups across a variety of practice settings, including small community and larger academic sites in a large city, as represented in this study [36-38].

### Action Items

The overlap of PositiveLinks functionalities with identified patient needs and facilitators of care, as well as its ability to further address identified barriers to care, is critical to emphasize to enhance stakeholder buy-in and, ultimately, uptake upon dissemination of the PositiveLinks app. Addressing features of PositiveLinks that are redundant with existing clinic technologies prevents provider technology fatigue, informing the selection of features for the PositiveLinks app tailored for the DC Cohort context. By further demonstrating the additional features and advantages PositiveLinks has over currently used technology (eg, social support aspects of the community board and easy connection to local and regional resources), we hope to encourage uptake of the app. For example, PositiveLinks may be able to add value by being easy to use and convenient, providing at-hand access, and having appropriate security to relieve privacy concerns.

Providers highlighted concerns related to perceptions about the additional time that PositiveLinks could incur on already busy days, as well as potential issues related to security, clinic workflow, and institutional approvals. To address these concerns, training will include security measures within the PositiveLinks app as well as how various functionalities may support the staff’s current workflow. For example, retention activities already conducted by patient outreach teams can be facilitated with the direct provider-patient messaging feature, or updated eligibility applications managed by clinic staff can be expedited using the document upload feature. The research team will be available to assist and provide all the required documentation to support the technical approval process for each site.

Several suggestions for tailored building were proposed by patients and providers, with additional resources and reminders being the most suggested items. We have worked with each of the PositiveLinks intervention sites to assist in compiling a comprehensive and site-specific list of resources. Potential clinic-specific documents could include local food bank resources, DC Metro schedule and information, and patient forms. Although the DC area has a wide array of services, navigating and sorting through services can be challenging for patients [8]. By working with clinic sites to select those most relevant to that location or patient population, we hope to increase access to existing services. Patients expressed interest in additional reminders to support more comprehensive self-management, such as weight tracking or reminders for additional non–HIV-related medications. Although these were considered, some of the suggestions were outside the scope of PositiveLinks and will not be able to be implemented for the trial.

**Figure 2:**
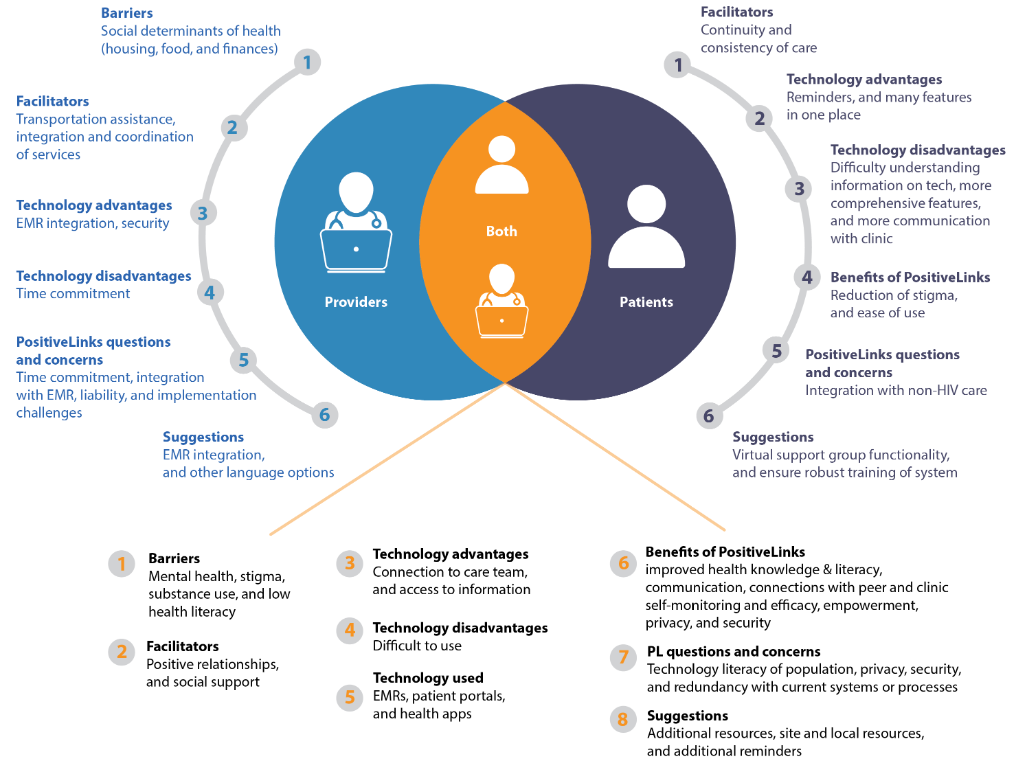
Comparison diagram between patient and provider perspectives. EMR: electronic medical record; PL: PositiveLinks.

As the PositiveLinks platform is a tool for both providers and patients, we ensured that these key stakeholders were able to express their likes, dislikes, concerns, and suggestions. Figure 2 depicts the similarities and differences between the patient and provider views on the barriers to and facilitators of engagement, technical advantages and disadvantages of their currently used systems (EMRs), anticipated benefits of PositiveLinks, PositiveLinks-related questions and concerns, and PositiveLinks-related suggestions. Understanding the different viewpoints and needs of these 2 groups was important for tailoring an app that met the needs of both.

This study was conducted in the midst of the COVID-19 pandemic, when providers and patients were learning to transition to care in a virtual form. Despite the barriers the pandemic generated, we found that both patients and providers highlighted mental health challenges, substance use, and stigma as major barriers to retention in care and viral suppression, whereas providers also frequently mentioned social determinants of health as barriers. We observed that patients tended to favor adding functionalities to PositiveLinks to further support their treatment goals in a more holistic fashion, whereas provider perspectives involved considerations related to PositiveLinks’ impact on clinic workflow and efficiency and ensuring that functionalities did not increase provider burden. Ultimately, both interests had to be considered when developing the final build to encourage engagement from both user groups.

### Strengths

Our formative development process has several strengths. We were able to gather qualitative data from 2 groups representing a diverse set of interests and perspectives, which could then be applied directly toward building an app that could meet the needs of both groups. All qualitative data were double coded to ensure that the codes and themes were applied in a standardized fashion. A coding agreement of ≥70% was achieved for the transcripts. African American individuals have historically been underrepresented in medical and mHealth research [39,40]. Most patient participants in this study (29/32, 91%) identified as Black, which aligns with the populations most affected by HIV at the national level and at the local level in DC [41]. Participants in this formative study were also demographically representative (age, gender identity, and race) of the larger DC Cohort [42].

### Limitations

Although we collected data from both provider and patient participants, there were some limitations. The sample size for providers (n=29) and patient FGs (n=32) was moderate, and the latter represented patients already enrolled in the DC Cohort who might not be representative of all people with HIV in Washington, DC. Furthermore, no Spanish-language patient FGs were conducted as the study team members moderating sessions were limited to conducting the FGs in English because of time and logistic constraints.

This work revealed many opportunities to optimize the platform, but not all suggestions will be implemented because of various limitations on the feasibility of the suggested modifications. For example, both providers and patients suggested the integration of PositiveLinks into their HIV provider’s clinic EMR or for PositiveLinks to be linked to other specialty clinics, such as obstetrician gynecologist, nephrologist, or primary care provider clinics. Patients also wanted the ability to conduct virtual support groups via the PositiveLinks platform so that they could see and relate to local peers going through the same issues and struggles. Although we believe both suggestions are relevant, those modifications are not currently feasible given the amount of human resources, multiple system integrations, and security measures that would have to be committed to make them possible at the randomized controlled trial stage. If the randomized controlled trial demonstrates positive outcomes for the intervention sites compared with the control sites, the team will consider adding these features for a future implementation project.

### Next Steps

Following this formative study, we worked with our software development team to incorporate suggestions and the finalized set of features into the PositiveLinks app for the DC Cohort. We customized each individual clinic resource page within PositiveLinks to show clinic-, local-, and region-specific information. After the initial tailoring, we performed beta testing of the app with 1 to 2 participants from each clinic (n=12) to further iterate the app and identify any technical challenges for troubleshooting. Beta testing included purposive sampling of at least 2 native Spanish speakers.

### Conclusions

PositiveLinks has been associated with positive health outcomes for people with HIV; however, it has not been specifically adapted for use in an urban population in the United States. Moreover, Black people are underrepresented in mHealth research. Our formative work provides insights into the adaptations needed to tailor the app for use in DC and for a predominantly Black population of people with HIV. By conducting formative research with both patients and providers, we aim to tailor the app for optimal uptake and use by both stakeholder groups. These findings will be used to help inform the building to be used in beta testing of the app, followed by dissemination of the app in our cluster randomized controlled trial, launched in November of 2022.

## Abbreviations

COHAH: District of Columbia Regional Planning Commission on Health and HIV
DC: District of Columbia
EMR: electronic medical record
FG: focus group
IRB: institutional review board
mHealth: mobile health
PI: principal investigator
REDCap: Research Electronic Data Capture

## Data Availability

The data sets generated and analyzed during this study are available from the corresponding author upon request.
